# Previous oil exposure alters Gulf Killifish *Fundulus grandis* oil avoidance behavior

**DOI:** 10.7717/peerj.10587

**Published:** 2020-12-18

**Authors:** Charles W. Martin, Ashley M. McDonald, Guillaume Rieucau, Brian J. Roberts

**Affiliations:** 1 UF/IFAS Nature Coast Biological Station, University of Florida, Cedar Key, FL, United States of America; 2 Louisiana Universities Marine Consortium, Chauvin, LA, United States of America

**Keywords:** Macondo, Deepwater Horizon, Fish, Gulf of Mexico, Salt marsh, Hydrocarbon

## Abstract

Oil spills threaten the structure and function of ecological communities. The *Deepwater Horizon* spill was predicted to have catastrophic consequences for nearshore fishes, but field studies indicate resilience in populations and communities. Previous research indicates many marsh fishes exhibit avoidance of oil contaminated areas, representing one potential mechanism for this resilience. Here, we test whether prior oil exposure of Gulf killifish *Fundulus grandis* alters this avoidance response. Using choice tests between unoiled and oiled sediments at one of three randomized concentrations (low: 0.1 L oil m^−2^, medium: 0.5 L oil m^−2^, or high: 3.0 L oil m^−2^), we found that, even at low prior exposure levels, killifish lose recognition of oiled sediments compared to control, unexposed fish. Preference for unoiled sediments was absent across all oil concentrations after oil exposure, and some evidence for preference of oiled sediments at high exposure was demonstrated. These results highlight the lack of response to toxic environments in exposed individuals, indicating altered behavior despite organism survival. Future research should document additional sublethal consequences that affect ecosystem and food web functioning.

## Introduction

The 2010 *Deepwater Horizon* (DwH) oil spill in the Gulf of Mexico (USA) impacted nearshore ecosystems from the Louisiana coast to the Florida Panhandle (Michel et al., 2013). Over 87 days, approximately 4.9 million barrels of oil (McNutt et al., 2012) ultimately covered an estimated 180,000 km^2^ of Gulf waters and over 1,100 km of coastal wetlands with ∼95% of these being in Louisiana (Mitra et al., 2012; Michel et al., 2013; Nixon et al., 2016). The spill predominantly impacted salt marshes in nearshore areas, with significant economic implications given they serve as key habitats for the young of many commercial and recreational fishery species (Peterson & Turner, 1994; Lellis-Dibble, McGlynn & Bigford, 2008; Rozas, Martin & Valentine, 2013; Baker et al., 2020). To date, field assessments of coastal fish populations and communities have shown resistance and resilience to, and in some cases rapid recovery from, the toxic effects of oil (Moody, Cebrian & Heck, 2013; Fodrie et al., 2014; Able et al., 2015; Schaefer, Frazier & Barr, 2016). Several potential explanations have been given for the unanticipated lack of severe impacts to populations/communities in nearshore ecosystems (Fodrie et al., 2014), including behavioral emigration from oiled areas (Martin, 2017), sublethal impacts to individuals (Whitehead et al., 2012; Dubansky et al., 2013) that do not translate to higher levels of organization, indirect food web mechanisms that provide predator release and/or stimulation of production (McCann et al., 2017), and cessation of fishing (Fodrie & Heck, 2011; Schaefer, Frazier & Barr, 2016; Martin et al., 2020).

Studies across a wide range of organisms, from zooplankton (Seuront, 2010) to marine mammals (Smultea & Würsig, 1995; Ackleh et al., 2012), have indicated complex recognition patterns and behavioral avoidance of oiled conditions. For example, calanoid copepods alter swimming behavior to avoid water-soluble diesel oil to limit exposure (Seuront, 2010). At larger spatial scales, sperm whales relocated from their historically occupied areas due to the DwH oil spill (Ackleh et al., 2012). Dolphins exhibit similar avoidance responses (Smultea & Würsig, 1995) and have been trained in the detection of oil (Geraci, St Aubin & Reisman, 1983). Conversely, American lobsters have shown attraction to hydrocarbons such as kerosene (Atema, 1976), which has been used as a bait by commercial fishermen.

Fish behavior is also known to be affected by chemical pollutants (Saaristo et al., 2018; Jacquin et al., 2020). Diverse taxonomic groups have demonstrated strong behavioral responses to acute crude oil contamination (Weis et al., 2001; Martin, 2017; Schlenker et al., 2019a). Freshwater fishes such as fathead minnows *Pimephales promelas* (Farr, Chabot & Taylor, 1995), rainbow trout *Oncorhynchus mykiss* (Carr et al., 1990), pink salmon fry *Oncorhynchus gorbuscha* (Rice, 1973), Caspian roach *Rutilus caspicus* (Lari et al., 2015), and striped bass *Morone saxatilis* (Carr et al., 1990) avoid hydrocarbon contaminated areas, albeit at different thresholds and concentrations. Estuarine and marine fishes such as flatfishes (Moles, Rice & Norcross, 1994), juvenile spot *Leiostomus xanthurus* (Hinkle-Conn et al., 1998), European seabass *Dicentrarchus labrax* (Claireaux et al., 2004), and mahi-mahi *Coryphaena hippurus* (Schlenker et al., 2019a) exhibit an avoidance response to the toxic chemical contaminants found in petroleum hydrocarbons.

In salt marshes, previous work (Martin, 2017) has demonstrated that ecologically important fishes such as Gulf killifish *Fundulus grandis*, sailfin molly *Poecilia latipinna*, and sheepshead minnow *Cyprinodon variegatus* avoid oil contaminated sediments, but display a reduced response to weathered oil indicating they likely react to the volatile, aromatic compounds that are lost as oil degrades and weathers due to a combination of factors including UV exposure (Bacosa, Erdner & Liu, 2015), wave action (Daling et al., 2014), microbial processing (Hazen et al., 2010), among others. Shallow, soft-sediment areas dominate the inshore reaches of the northern Gulf of Mexico (Connor & Day, 1987) and benthic organisms living within and on these sediments serve as important food sources for numerous species, including one of the most abundant Gulf coast marsh species *F. grandis*. For example, Rozas & LaSalle (1990) reported that the major dietary constituents of *F. grandis* are found associated with these sediments: fiddler crabs, amphipods, and hydrobiid snails. As such, recognition of the quality and contamination of these habitats is critical for *F. grandis* as these areas are linked with successful foraging and fitness.

Previous research indicates that oil exposure can alter fish detection of oil. For example, exposure to the water-accommodated fraction of oil in Atlantic stingrays *Hypanus sabinus* damaged olfactory function (Cave & Kajiura, 2018). After oil exposure, bicolor damselfish *Stegastes partitus* showed a reduction in the response to conspecific alarm cues (Schlenker et al., 2019b). This damage to olfactory mechanisms or central nervous processing may further reduce an organism’s capacity to detect and respond to oil contamination (sensu Schlenker et al., 2019a).

Here, we present the results of a series of experiments to test the effects of previous oil exposure on oil-contaminated sediment avoidance in salt marsh fish. Using the Gulf killifish *F. grandis*, a widely-used sentinel species for toxicological and ecological studies (Able et al., 2015; Vastano et al., 2017; Jensen et al., 2019), we exposed animals to a range of concentrations in experimentally oiled marsh conditions and then subsequently tested behavioral avoidance patterns in simple choice tests. The overarching objective of this project was to determine if prior exposure influences avoidance behavior and, if so, to identify what exposure level influences these changes. Evidence that even the lowest sublethal levels of hydrocarbon exposure result in non-avoidance of contaminated sediments could suggest there are behaviorally-associated impacts to overall fitness or survival of these ecologically important marsh fish.

## Materials & Methods

### Fish exposure

Fish were exposed to oil for 10–15 days in experimentally oiled marsh mesocosms at the Louisiana Universities Marine Consortium in Cocodrie, LA during August/September 2019. This exposure period is based on studies of known site fidelity in *F. grandis* (Nelson, Sutton & DeVries, 2014; Jensen et al., 2019). Briefly, we utilized 12 hydrologically independent *Spartina alterniflora* marsh mesocosms (3.05 m diameter, 1.83 m height) each with its own paired tidal surge tank generating daily tidal cycles with range of 25 cm (flooding marsh ∼10 cm at high tide) via a water control system of blowers and airlifts (Alt, 2019). During flooded marsh conditions (∼40% of the time), fish had access to ∼7.3 m^2^ of marsh platform (∼10 cm water depth at high tide) and the adjacent deeper water (∼40 cm deep at high tide) in the surrounding trough. When water was off the marsh, fish were restricted to ∼1.4 m^2^ of surface area in the trough with water depths as low as 15 cm at low tide. Intact salt marsh plugs (30 cm diameter ×  50 cm depth) at natural densities from nearby unoiled marshes were established in mesocosms approximately 18 months prior to oiling. Light Louisiana Sweet (LLS) blended crude oil, API Gravity 40.1, similar to that which was released in the DwH oil spill, was acquired from Placid Refining Company LLC in May 2018. This oil was evaporatively weathered by 30% of its volatile components, as measured by gas chromatography, using a nitrogen gas sparging system placed in the barrel of liquid oil as received from Placid over a period of 150 days. The bubbling system not only expedited evaporation, but also mixed the contents of the barrel to ensure conformity of its contents. A single application was applied to the water at uniformly spaced locations at high tide to each mesocosm on July 8, 2019 at one of four concentrations, scaling roughly to Shoreline Cleanup and Assessment Team (SCAT) categories observed on shorelines after the DwH spill (Michel et al., 2013; Nixon et al., 2016): control/no oil (0.0 L oil m^−2^), low (0.1 L oil m^−2^), medium (0.5 L oil m^−2^), and high (3.0 L oil m^−2^) oil concentrations. Additional details on the mesocosm setup can be found at https://robertsresearchlab.weebly.com/mesocosms.html.

We captured *F. grandis* from the nearby salt marsh using baited minnow traps and held them in a separate 450 liter aquarium for 2-4 days to minimize any mortality due to handling before being introduced to the 15 cm wide trough surrounding plants within each mesocosm. During low tide, fish were restricted to a 15 cm deep water column and at high tide events fish gained access to oiled or unoiled experimental marsh platforms (at a water depth of ∼10 cm) to forage. A total of 18 fish were added to each mesocosm between 22 August (12 fish added) and 27 August 2019 (6 additional fish added to increase available fish for the avoidance experiment due to mortality observed after the first few days). A total of 54 fish/treatment (18 fish in three mesocosm of each oil treatment) were introduced to mesocosms and recaptured prior to experiments using dip nets. Fish were exposed to oil treatments for 10 (27 August additions) to 15 (22 August additions) days with oil having been further weathered in the mesocosms for 45 to 60 days prior to initiation of the experiments described here. The mean ± standard error surface soil (0–5 cm) total petroleum hydrocarbon (TPH) concentrations in the high oil treatments (419 ± 24 mg/g soil) were ∼10 and ∼40 times higher than in the moderate (39 ± 5 mg/g soil) and low (10 ± 0.4 mg/g soil) oil treatments (mean of 19 August and 9 September samplings; E. Overton & B. J. Roberts, 2020, unpublished data). These concentrations are similar to those found in Louisiana salt marsh field conditions (Lin et al., 2016). Only survivors from these exposures were used in the subsequent experiment to measure sublethal effects, and they were held for 48 h in separate aquaria containing unoiled seawater by treatment prior to use in behavioral experiments to ensure no additional mortality. Seawater used for holding fish and in the avoidance experiment (described below) was passed through an ultraviolet filter then passed through a 0.2 µm filter to remove any background oil that may confound results.

### Avoidance experiment

To test the behavioral response of fish exposed to different oil concentrations, we used a choice test following the design reported in Martin (2017). Thirty-eight-liter aquaria were filled with 3 L total of clean, unoiled sediment to a depth of 18 cm. Each aquarium offered a choice between unoiled and oiled sediment, randomized on each side of the aquaria to reduce the chances of any unknown external cues affecting fish behavior. In addition, we conducted trials with no oil on either side of the aquarium and found no preference; these data were not used in further analyses. Concentrations followed previous experiments (Martin, Hollis & Turner, 2015; Martin, 2017), and fish were given a choice between no oil and low oil (10 mL oil per L sediment), no oil and medium oil (20 mL oil per L sediment), or no oil and high oil (40 mL oil per L sediment) contamination. In these tests, we used 25% weathered oil (from the same source barrel as used in the larger mesocosm experiment) as this was representative of what came inshore (Reddy et al., 2012) and is the same degree of weathering used in previous experiments (Martin, 2017). Using gloves, we hand-mixed sediment (both with or without oil) on the randomized side of the aquaria at the assigned concentration and a thin layer (approximately 2 cm deep) of unoiled sand was placed uniformly on top to prevent the oil/sand mixture from floating and affecting the adjacent, unoiled side of the aquaria. Using only sediment in tanks prevented confounding effects of vegetation due to structural refuge.

All *F. grandis* used in this study were adult individuals between 57 mm and 105 mm and used only once in a trial. Mortality during the exposure duration in oiled mesocosms limited the number of available fish, and as a result we replicated most comparisons 8 times, except for medium exposure fish (no oil vs medium oil was replicated 4 times) and high exposure fish (only no vs low oil, the most conservative of the oil level options, was tested with 8 replicates to avoid a lower sample size that would contribute to an inability to statistically detect trends). We held salinity constant at 7.0 PSU and temperature ranged from 26.8–28.6 °C during trials (both comparable to the conditions in the mesocosms at the time of collection).

For each trial, one fish from a randomized exposure was introduced to the aquarium, allowed a 5-minute acclimation period, and its movements between the two sides of the aquarium recorded using a GoPro camera over the course of 10 min. This trial period mimics previous fish behavioral experiments (Gerlach et al., 2007; Paris et al., 2013; Martin, 2017). The side of the aquarium occupied by fish (no oil or assigned oil treatment) was recorded by analyzing a frame systematically every 30 s over the trial period and noting fish position within the aquarium. The proportion of time in each side was then calculated as the number of observations taken on that side divided by the total observations.

Data were statistically analyzed in 2 ways, identical to analyses performed in Martin (2017): (1) to determine whether fish deviated from an expected 1:1 occupancy pattern, we conducted a paired *t*-test for each previous exposure level and oil vs no oil comparison (Peterson & Renaud, 1989) and (2) to compare differences across treatments, we analyzed proportion of time spent in oil using a general linear model (GLM) with factors of previous exposure and oil vs. no oil comparison. Tukey’s post hoc test was used to determine significant pairwise differences. Assumptions (normality and homogeneity of variance) were tested for all comparisons and nonparametric alternatives (signed rank test) used if transformations failed to meet assumptions and considered results significant at *p* < 0.05. To graphically display data, a ratio of the number of times fish occupied each side of the aquarium was generated and plotted, such that deviation below 1 indicates avoidance of oil and above the line denotes preference for oil.

### Ethics statement

All field collections were made under Louisiana Department of Wildlife and Fisheries Scientific Collecting Permit # SCP 200. The use of vertebrate organisms was conducted with IACUC approval and staff training from University of Florida under protocol 201710044. As the goal of the study was to measure sublethal effects of oil on fish behavior, humane endpoints were not used and were not possible during the 10–15 day exposure, as fish were released into turbid mesocosms and unable to be monitored. Moreover, analgesics and anaesthetics were not used because of the alterations to behavior that we were quantifying.

## Results

**Figure 1:**
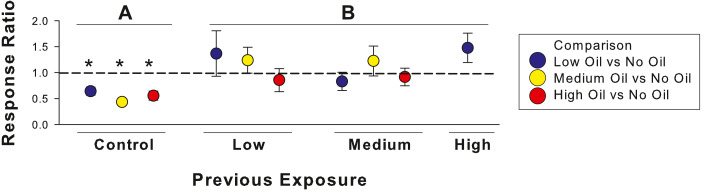
Ratio of time spent in oil to time spent in control side of tank across previous exposures. Dashed line indicates 1:1 (no preference), with values >1 indicating more time over oiled sediments and <1 indicating less time over oiled sediments. Colors represent values for preference comparisons between no oil and low (blue), medium (yellow), and high (red) oil concentrations. Asterisks indicate fish deviate from an expected 1:1 occupancy pattern (Table 1) and different letters indicate statistical differences among treatments.

Previous exposure influenced fish preference patterns for oil contaminated sediments (Fig. 1, Table 1). Unexposed (control) fish showed significant avoidance of the oiled side of aquaria, regardless of the oil concentration choice given (Table 1). After exposure to oil, even at low concentrations, this avoidance response disappeared. Fish unexposed to oil spent on average 66% of time over uncontaminated sediments, a trend that decreased with previous exposure to low (52%), medium (55%), and high (44%) concentrations. At high previous exposure concentrations, we noted 7 of the 8 fish spent more time over oiled sediments in the aquaria, although the time spent on the oiled side was not significant (Table 1).

**Table 1:** Statistical results (either paired *t*-test or signed rank test) for each previous exposure and comparison. Control fish preferred no oil, a response which is lost even at low previous oil exposure. Significant *p*-values are shown in bold.

**Previous Exposure**	**Comparison**	**Statistical Test**	**N**	**Test Statistic^a^**	*p*
Control (No Oil)	Low Oil vs No Oil	Paired *t*-test	8	−4.204	**0.004**
	Medium Oil vs No Oil	Paired *t*-test	8	−7.105	**<0.001**
	High Oil vs No Oil	Signed rank	8	2.388	**0.016**
Low Oil	Low Oil vs No Oil	Paired *t*-test	8	<0.001	1.000
	Medium Oil vs No Oil	Paired *t*-test	8	0.235	0.821
	High Oil vs No Oil	Signed rank	8	1.550	0.148
Medium Oil	Low Oil vs No Oil	Paired *t*-test	8	−1.546	0.166
	Medium Oil vs No Oil	Paired *t*-test	4	0.378	0.731
	High Oil vs No Oil	Paired *t*-test	8	−1.080	0.316
High Oil	Low Oil vs No Oil	Paired *t*-test	8	1.418	0.199

**Notes.**

a Test statistic: T for Paired *t*-test, *Z* for Signed Rank test

The oil concentration used in the choice test had a smaller influence on fish behavior than previous oil exposure. Results of the GLM confirmed these results, which indicated that previous exposure (*F*_3,70_ = 6.83, *p* < 0.001), not oil concentration in preference test (*F*_2,70_ = 0.38, *p* = 0.684), drove behavioral patterns. Pairwise comparisons indicated that control, unexposed fish had significantly stronger avoidance of oil than fish previously exposed to low (*p* = 0.0034), medium (*p* = 0.0337), or high (*p* = 0.0029) oil concentrations, with low, medium, and high not significantly different from each other (*p* > 0.05).

## Discussion

The 2010 DwH oil spill was an unprecedented stressor for northern Gulf ecosystems, with oil impacting emergent and submerged plants (Lin & Mendelssohn, 2012; Silliman et al., 2012), invertebrates (McCall & Pennings, 2012; Powers et al., 2017; Zengel et al., 2016), and fishes (Fodrie & Heck, 2011; Able et al., 2015; Schaefer, Frazier & Barr, 2016). While the full effect on the nearshore food web may not yet be fully realized because of the numerous and complex indirect food web mechanisms (McCann et al., 2017; Barron et al., 2020), many studies have documented significant impacts to molecular, genomics, and development of fishes (Whitehead et al., 2012; Dubansky et al., 2013) and resilience in populations and communities to oil’s toxic effects (Fodrie et al., 2014; Martin et al., 2020). Among the proposed explanations for this resilience, despite oil’s known toxicity, is the behavioral emigration of organisms at small spatial scales to avoid exposure to contamination (Martin, 2017). Here, we demonstrate that exposure to oiled marshes, even at low concentrations of weathered oil (0.1 L oil m^−2^), can impact a common marsh fish’s ability to avoid oil contaminated sediments.

A further exploration of the current dataset indicated one anomalous trial in high exposure fish. In this trial, the fish spent 65% of time over unoiled sediments. With the exception of this trial, fish exposed to high oil concentrations spent 60% of time over oil sediments (range: 50–75%), a significant preference (*t*(6) = 2.517; *p* = 0.045) for oiled sediments at low concentration over unoiled sediments (the only test conducted on high exposure fish as a conservative estimate due to lack of experimental organisms). We hypothesize that this apparent preference for oiled sediments could be due to relaxation of normal physiological functioning causing an anaesthetic effect (Barron et al., 2004). Several studies have documented a narcotic effect on fishes due to chronic exposure to oil (Barron et al., 2004; Incardona, Collier & Scholz, 2004). This can result in a nervous system sedation (Lin & Tjeerdema, 2008), as well as increased respiratory rate (Brocksen & Bailey, 1973) and decreased swimming performance (Stieglitz et al., 2016). These effects can be reversed, however, usually on the order of days after exposure has been removed (Brocksen & Bailey, 1973). In this experiment, we only conducted choice trials at low concentrations for the high exposure treatments, leaving open the possibility that a stronger response could have been observed had more fish survived the exposure duration. As the goal of this study was to document the alterations to *F. grandis* behavior and not to test the physiological mechanisms driving the response to oil, we can only speculate that there could be acclimation or damage to olfactory or other organs (Cave & Kajiura, 2018; Schlenker et al., 2019a) based on our experiments.

Previous exposure to oil is known to have many negative consequences for individuals. For example, unexposed mahi-mahi avoided higher concentrations of water accommodated fraction of oil, but exposed individuals demonstrated a lack of response (Schlenker et al., 2019a). In this case, the authors explicitly tested for, and did not find, damage to olfactory acuity from oil exposure. In other studies, damage to the higher order central nervous system processing was implicated in this decreased oil avoidance behavior. Olfactory damage is also known to occur with oil exposure in some fishes (such as Atlantic stingrays, Cave & Kajiura, 2018) and reduced recognition of threats via these cues is also possible (Schlenker et al., 2019b). The specific chemical constituents in weathered oil involved in these detrimental physiological changes remain unclear. Given that weathered oil comprised the bulk of the oil that came ashore (Reddy et al., 2012), it is likely that, based on field abundance comparisons (Fodrie & Heck, 2011; Able et al., 2015; Martin et al., 2020) fish survival may have been higher than expected and thus sublethal effects may constitute the largest impact on marsh organisms. In our exposures, we noted mortality and lack of recapture for some treatments, particularly in medium and high oil mesocosms, which precluded the full range of preference tests in the avoidance experiment. Specifically, out of the original 54 fish released we recaptured 54, 34, 24, and 10 fish in control, low, medium, and high treatments, respectively. These differences in mortality corresponded to survivorship of 100% (control), 62.0% (low), 44.4% (medium), and 18.5% (high) in mesocosm treatments. Importantly, fish were held for 48 h post-exposure prior to use in behavioral assays and no additional mortality was detected during this period or during behavioral trials. We acknowledge this mortality and lack of recapture resulted in low replication for some treatments (medium exposure and no oil vs. medium oil choice test) and lack of comparisons for others (high exposure). As a result, findings from the lower replication trials should be considered preliminary and serve as a template for future studies to address this deficiency. Without a post-mortem examination, we cannot definitively state whether killifish mortality during the mesocosm exposure duration was caused by toxicity or because of behavioral responses to weathered oil exposure (e.g., foraging inefficiencies). As such, these findings support the notion that even if fish survive oil exposure there are significant behavioral responses that might influence their long-term survival.

The sublethal effects of the DwH oil spill have remained, for many organisms, largely unexplored in the decade following the spill (but see Rozas, Minello & Miles, 2014; Cave & Kajiura, 2018; Martin & Swenson, 2018; Schlenker et al., 2019a; Schlenker et al., 2019b). Given the importance of physiological processes such as sensory mechanisms and olfaction for many critical activities, such as foraging (Webster et al., 2007; Johannesen, Dunn & Morrell, 2012), habitat recognition (Benfield & Aldrich, 1992; Forward et al., 2003), and predator avoidance (Dixson, Munday & Jones, 2010; Martin et al., 2010), it is possible that these and other sublethal effects resulting from oil exposure may have great consequences. Previous studies have indicated that oil can have other important sublethal effects on fishes and invertebrates. For example, oil presence triggered a 60% decrease in penaeid shrimp *Farfantepenaeus aztecus* growth rate (Rozas, Minello & Miles, 2014) and foraging by darter gobies *Gobionellus boleosoma* can change 50–100% in sediments highly contaminated with diesel fuel (Gregg, Fleeger & Carman, 1997). Spot *L. xanthurus* do not alter feeding behavior at moderate-high concentrations of diesel oil suggesting continued exposure while feeding on benthic organisms may occur (Hinkle-Conn et al., 1998). Given the known deleterious impacts to other fishes, we anticipate that similar sublethal consequences were present in marsh fishes after DwH, but have remained understudied. We propose that additional research on the sublethal effects of oil (including impacts to top down control via predation or predator release) need to be conducted to gain a broader understanding of the full scope of DwH damages to northern Gulf of Mexico ecosystems.

Oil released from the DwH drilling rig was burned at the surface, collected on the water or as it came ashore on wetlands and beaches, and chemically dispersed using Corexit^®^ dispersant (Peterson et al., 2012; Michel et al., 2013; Nixon et al., 2016). Wetlands accounted for over half of the oiled shoreline (∼1,100 of ∼2,100 km), with >95% of oiled marshes in Louisiana (Nixon et al., 2016). However, much of the oil remains unaccounted for (McNutt et al., 2012) and is thought to reside in sediments throughout the region. Previous spills such as the *Exxon Valdez* (Renner et al., 2006; Li & Boufadel, 2010), *Florida* barge in Massachusetts (Culbertson et al., 2008), and *Ixtoc-I* (Schrope, 2010) all indicate that oil can persist buried in the sediment where oil weathering rates are low (Boufadel et al., 2010). Thus, marsh fishes may be vulnerable to sublethal oil exposure and the loss of avoidance behaviors after exposure in marsh species may have more subtle, but still substantial, implications for the marsh food web long after the oiling event. Unlike in pelagic species where exposure is comparatively more limited because oil moves long distances across the surface with currents and wind, weathers, biodegrades, or sinks to deep sediments, once oil reaches marsh sediments exposure may be extended. Once exposed, these marsh fishes lose recognition and remain vulnerable to oil contamination in the short or long term as oil gets trapped by the plants and buried or slowly degraded over time. Enhancement of erosion rates (Silliman et al., 2012; Martin, Hollis & Turner, 2015; Turner, McClenachan & Tweel, 2016) and sediment remobilization after large storm events, such as the frequent Gulf of Mexico hurricanes (Khanna et al., 2013; Michel et al., 2013), may re-expose remaining oil to saltmarsh flora and fauna, continuing to sublethally impact organisms for decades to come. Many resident and transient species spend some part of their life cycles in these contaminated areas and could be impacted for sustained periods, necessitating the need for continued study of oil impacts and population trends in these vital ecosystems.

## Conclusions

We tested whether prior exposure to oil alters the Gulf killifish’s avoidance response of oil. Given the limitations and ethics of experimentally oiling field locations, we used an ongoing mesocosm experiment to expose individuals to oil. After a short 48-hr holding period to ensure no additional mortality due to the exposure duration, fish avoidance of oil was then tested in simple choice tests. We found that, even at low oil exposure levels, fish lost their response to oil compared to unexposed, control fish. This research suggests that fish surviving short-term exposure durations may continue to incur sublethal effects.

##  Supplemental Information

10.7717/peerj.10587/supp-1 Supplemental Information 1 Dataset Each row is data from one trial measuring fish occupancy in the tank. Treatment-Previous Exposure column is the level of previous exposure (control, low, medium, or high) that fish were exposed to; Comparison-Oil Used in Preference Test: refers to the relative oil concentration used in the preference experiments (Low, Medium, High); Proportion of Time in Oil: proportion calculated from observations of location preference made every 30 s for 10 min trial duration divided by total number of observations; Proportion of Time in No Oil: proportion calculated from observations of location preference made every 30 s for 10 min trial duration divided by total number of observations.

## Additional Information and Declarations

### Field Study Permissions

The following information was supplied relating to field study approvals (i.e., approving body and any reference numbers): All field collections were made under Louisiana Department of Wildlife and Fisheries Scientific Collecting Permit # SCP 200.

### Data Availability

The following information was supplied regarding data availability: Data are publicly available through the Gulf of Mexico Research Initiative Information & Data Cooperative (GRIIDC): Martin, C.W., Roberts, B.J. 2020. Laboratory experiments to determine effects of previous exposure to oil on Fundulus grandis avoidance behavior. Distributed by: Gulf of Mexico Research Initiative Information and Data Cooperative (GRIIDC), Harte Research Institute, Texas A&M University–Corpus Christi. doi:https://doi.org/10.7266/n7-3qjh-2f11.

## References

[ref-1] Able KW, López-Duarte PC, Fodrie FJ, Jensen OP, Martin CW, Roberts BJ, Valenti J, O’Connor K, Halbert SC (2015). Fish assemblages in Louisiana salt marshes: effects of the Macondo oil spill. Estuaries and Coasts.

[ref-2] Ackleh AS, Ioup GE, Ioup JW, Ma B, Newcomb JJ, Pal N, Sidorovskaia NA, Tiemann C (2012). Assessing the Deepwater Horizon oil spill impact on marine mammal population through acoustics: endangered sperm whales. The Journal of the Acoustical Society of America.

[ref-3] Alt DC (2019). An airlifted tidal mesocosm for oil degradation studies. LSU Doctoral Dissertations.

[ref-4] Atema J (1976). Sublethal effects of petroleum fractions on the behavior of the lobster, *Homarus americanus*, and the mud snail, *Nassarius obsoletus*. Estuarine Processes.

[ref-5] Bacosa HP, Erdner DL, Liu Z (2015). Differentiating the roles of photooxidation and biodegradation in the weathering of Light Louisiana Sweet crude oil in surface water from the Deepwater Horizon site. Marine Pollution Bulletin.

[ref-6] Baker R, Taylor MD, Able KW, Beck MW, Cebrian J, Colombano DD, Connolly RM, Currin C, Deegan LA, Feller IC, Gilby BL, Kimball ME, Minello TJ, Rozas LP, Simenstad C, Turner RE, Waltham NJ, Weinstein MP, Ziegler SZ, zuErmgassen PSE, Alcott C, Alford SB, Barbeau MA, Crosby SC, Dodds K, Frank A, Goeke J, Gaines LA, Hardcastle FE, Henderson CJ, James WR, Kenworthy MD, Lesser J, Mallick D, Martin CW, McDonald AE, McLuckie C, Morrison BH, Nelson JA, Norris GS, Ollerhead J, Pahl JW, Ramsden S, Rehage JS, Reinhardt JF, Rezek RJ, Risse LM, Smith JAM, Sparks EL, Staver LW (2020). Fisheries rely on threatened salt marshes. Science.

[ref-7] Barron MG, Carls MG, Heintz R, Rice SD (2004). Evaluation of fish early life-stage toxicity models of chronic embryonic exposures to complex polycyclic aromatic hydrocarbon mixtures. Toxicological Sciences.

[ref-8] Barron MG, Vivian DN, Heintz RA, Yim UH (2020). Long-term ecological impacts from oil spills: comparison of exxon valdez, hebei spirit, and deepwater horizon. Environmental Science & Technology.

[ref-9] Benfield M, Aldrich D (1992). Attraction of postlarval *Penaeus aztecus* Ives and *P. setiferus* (L.) (Crustacea:Decapoda:Penaeidae) to estuarine water in a laminar-flow choice chamber. Journal of Experimental Marine Biology and Ecology.

[ref-10] Boufadel MC, Sharifi Y, Van Aken B, Wrenn BA, Lee K (2010). Nutrient and oxygen concentrations within the sediments of an Alaskan beach polluted with the Exxon Valdez oil spill. Environmental Science & Technology.

[ref-11] Brocksen RW, Bailey HT (1973). Respiratory response of juvenile chinook salmon and striped bass exposed to benzene, a water-soluble component of crude oil. International oil spill conference, vol. 1973, no.1.

[ref-12] Carr RS, Barrows ME, Reichenbach NG, DeGraeve GM, Pollock TL, Fava JA, Glickman AH (1990). Investigation of preference-avoidance responses to an oil refinery effluent with striped bass and steelhead trout. Environmental Toxicology & Chemistry.

[ref-13] Cave EJ, Kajiura SM (2018). Effect of Deepwater Horizon crude oil water accommodated fraction on olfactory function in the Atlantic stingray, *Hypanus sabinus*. Scientific Reports.

[ref-14] Claireaux G, Désaunay Y, Akcha F, Aupérin B, Bocquené G, Budzinski H, Cravedi JP, Davoodi F, Galois R, Gilliers C, Goanvec C (2004). Influence of oil exposure on the physiology and ecology of the common sole *Solea solea*: experimental and field approaches. Aquatic Living Resources.

[ref-15] Connor WH, Day JW (1987). The ecology of Barataria Basin, Louisiana: an estuarine. Biological report 85.

[ref-16] Culbertson JB, Valiela I, Pickart M, Peacock EE, Reddy CM (2008). Long-term consequences of residual petroleum on salt marsh grass. Journal of Applied Ecology.

[ref-17] Daling PS, Leirvik F, Almås IK, Brandvik PJ, Hansen BH, Lewis A, Reed M (2014). Surface weathering and dispersibility of MC252 crude oil. Marine Pollution Bulletin.

[ref-18] Dixson DL, Munday PL, Jones GP (2010). Ocean acidification disrupts the innate ability of fish to detect predator olfactory cues. Ecology Letters.

[ref-19] Dubansky B, Whitehead A, Miller JT, Rice CD, Galvez F (2013). Multitissue molecular, genomic, and developmental effects of the Deepwater Horizon oil spill on resident Gulf killifish (*Fundulus grandis*). Environmental Science & Technology.

[ref-20] Farr AJ, Chabot CC, Taylor DH (1995). Behavioral avoidance of fluoranthene by fathead minnows (*Pimephales promelas*). Neurotoxicology and Teratology.

[ref-21] Fodrie FJ, Able KW, Galvez F, HeckJr KL, Jensen OP, López-Duarte PC, Martin CW, Turner RE, Whitehead A (2014). Integrating organismal and population responses of estuarine fishes in Macondo spill research. BioScience.

[ref-22] Fodrie FJ, Heck KL (2011). Response of coastal fishes to the Gulf of Mexico oil disaster. PLOS ONE.

[ref-23] Forward R, Tankersley R, Smith K, Welch J (2003). Effects of chemical cues on orientation of blue crab, *Callinectes sapidus*, megalopae in flow: implications for location of nursery areas. Marine Biology.

[ref-24] Geraci JR, St Aubin DJ, Reisman RJ (1983). Bottlenose dolphins, *Tursiops truncatus*, can detect oil. Canadian Journal of Fisheries and Aquatic Sciences.

[ref-25] Gerlach G, Atema J, Kingsford MJ, Black KP, Miller-Sims V (2007). Smelling home can prevent dispersal of reef fish larvae. Proceedings of the National Academy of Sciences of the United States of America.

[ref-26] Gregg JC, Fleeger JW, Carman KR (1997). Effects of suspended, diesel-contaminated sediment on feeding rate in the darter goby, *Gobionellus boleosoma* (Teleostei: Gobiidae). Marine Pollution Bulletin.

[ref-27] Hazen TC, Dubinsky EA, DeSantis TZ, Andersen GL, Piceno YM, Singh N, Jansson JK, Probst A, Borglin SE, Fortney JL, Stringfellow WT (2010). Deep-sea oil plume enriches indigenous oil-degrading bacteria. Science.

[ref-28] Hinkle-Conn C, Fleeger JW, Gregg JC, Carman KR (1998). Effects of sediment-bound polycyclic aromatic hydrocarbons on feeding behavior in juvenile spot (*Leiostomus xanthurus* Lacepede: Pisces). Journal of Experimental Marine Biology and Ecology.

[ref-29] Incardona JP, Collier TK, Scholz NL (2004). Defects in cardiac function precede morphological abnormalities in fish embryos exposed to polycyclic aromatic hydrocarbons. Toxicology and applied pharmacology.

[ref-30] Jacquin L, Petitjean Q, Côte J, Laffaille P, Jean S (2020). Effects of pollution on fish behavior, personality, and cognition: some research perspectives. Frontiers in Ecology and Evolution.

[ref-31] Jensen OP, Martin CW, Oken KL, Fodrie FJ, López-Duarte PC, Able KW, Roberts BJ (2019). Simultaneous estimation of dispersal and survival of the gulf killifish *Fundulus grandis* from a batch-tagging experiment. Marine Ecology Progress Series.

[ref-32] Johannesen A, Dunn AM, Morrell LJ (2012). Olfactory cue use by three-spined sticklebacks foraging in turbid water: prey detection or prey location?. Animal Behaviour.

[ref-33] Khanna S, Santos MJ, Ustin SL, Koltunov A, Kokaly RF, Roberts DA (2013). Detection of salt marsh vegetation stress and recovery after the Deepwater Horizon oil spill in Barataria Bay, Gulf of Mexico using AVIRIS data. PLOS ONE.

[ref-34] Lari E, Abtahi B, Hashtroudi MS, Mohaddes E, Døving KB (2015). The effect of sublethal concentrations of the water-soluble fraction of crude oil on the chemosensory function of Caspian roach, *Rutilus caspicus* (YAKOVLEV, 1870). Environmental Toxicology and Chemistry.

[ref-35] Lellis-Dibble KA, McGlynn KE, Bigford TE (2008). Estuarine fish and shellfish species in US commercial and recreational fisheries: economic value as an incentive to protect and restore estuarine habitat. NOAA Technical Document.

[ref-36] Li H, Boufadel MC (2010). Long-term persistence of oil from the Exxon Valdez spill in two-layer beaches. Nature Geoscience.

[ref-37] Lin CY, Tjeerdema RS, Jorgensen SE, Fath BD (2008). Crude oil, oil, gasoline and petrol. Encyclopedia of ecology, volume 1: ecotoxicology.

[ref-38] Lin Q, Mendelssohn IA (2012). Impacts and recovery of the *Deepwater Horizon* oil spill on vegetation structure and function of coastal salt marshes in the northern Gulf of Mexico. Environmental Science & Technology.

[ref-39] Lin Q, Mendelssohn IA, Graham SA, Hou A, Fleeger JW, Deis DR (2016). Response of salt marshes to oiling from the Deepwater Horizon spill: implications for plant growth, soil surface-erosion, and shoreline stability. Science of the Total Environment.

[ref-40] Martin CW (2017). Avoidance of oil contaminated sediments by estuarine fishes. Marine Ecology Progress Series.

[ref-41] Martin CW, Fodrie FJ, Heck KL, Mattila J (2010). Differential habitat use and antipredator response of juvenile roach (*Rutilus rutilus*) to olfactory and visual cues from multiple predators. Oecologia.

[ref-42] Martin CW, Hollis LO, Turner RE (2015). Effects of oil-contaminated sediments on submerged Vegetation: an experimental assessment of *Ruppia maritima*. PLOS ONE.

[ref-43] Martin CW, Lewis KL, McDonald AM, Spearman T, Alford SB, Christian R, Valentine JF (2020). Disturbance-driven changes to northern Gulf of Mexico nekton communities following the Deepwater Horizon oil spill. Marine Pollution Bulletin.

[ref-44] Martin CW, Swenson EM (2018). Herbivory of oil-exposed submerged aquatic vegetation *Ruppia maritima*. PLOS ONE.

[ref-45] McCall BD, Pennings SC (2012). Disturbance and recovery of salt marsh arthropod communities following BP Deepwater Horizon oil spill. PLOS ONE.

[ref-46] McCann MJ, Able KW, Christian RR, Fodrie FJ, Jensen OP, Johnson JJ, López-Duarte PC, Martin CW, Olin JA, Polito MJ, Roberts BJ (2017). Key taxa in food web responses to stressors: the Deepwater Horizon oil spill. Frontiers in Ecology and the Environment.

[ref-47] McNutt MK, Camilli R, Crone TJ, Guthrie GD, Hsieh PA, Ryerson TB, Savas O, Shaffer F (2012). Review of flow rate estimates of the Deepwater Horizon oil spill. Proceedings of the National Academy of Sciences of the United States of America.

[ref-48] Michel J, Owens EH, Zengel S, Graham A, Nixon Z, Allard T, Holton W, Reimer PD, Lamarche A, White M, Rutherford N (2013). Extent and degree of shoreline oiling: deepwater Horizon oil spill, Gulf of Mexico, USA. PLOS ONE.

[ref-49] Mitra S, Kimmel DG, Snyder J, Scalise K, McGlaughon BD, Roman MR, Jahn GL, Pierson JJ, Brandt SB, Montoya JP, Rosenbauer RJ (2012). Macondo-1 well oil-derived polycyclic aromatic hydrocarbons in mesozooplankton from the northern Gulf of Mexico. Geophysical Research Letters.

[ref-50] Moles A, Rice S, Norcross BL (1994). Non-avoidance of hydrocarbon laden sediments by juvenile flatfishes. Netherlands Journal of Sea Research.

[ref-51] Moody RM, Cebrian J, Heck KL (2013). Interannual recruitment dynamics for resident and transient marsh species: evidence for a lack of impact by the Macondo oil spill. PLOS ONE.

[ref-52] Nelson TR, Sutton D, DeVries DR (2014). Summer movements of the Gulf Killifish (*Fundulus grandis*) in a northern Gulf of Mexico salt marsh. Estuaries and Coasts.

[ref-53] Nixon Z, Zengel S, Baker M, Steinhoff M, Fricano G, Rouhani S, Michel J (2016). Shoreline oiling from the Deepwater Horizon oil spill. Marine Pollution Bulletin.

[ref-54] Paris CB, Atema J, Irisson JO, Kingsford M, Gerlach G, Guigand CM (2013). Reef odor: a wake up call for navigation in reef fish larvae. PLoS ONE.

[ref-55] Peterson CH, Anderson SS, Cherr GN, Ambrose RF, Anghera S, Bay S, Blum M, Condon R, Dean TA, Graham M, Guzy M (2012). A tale of two spills: novel science and policy implications of an emerging new oil spill model. BioScience.

[ref-56] Peterson CH, Renaud PE (1989). Analysis of feeding preference experiments. Oecologia.

[ref-57] Peterson GW, Turner RE (1994). The value of salt marsh edge vs interior as a habitat for fish and decapod crustaceans in a Louisiana tidal marsh. Estuaries.

[ref-58] Powers SP, Grabowski JH, Roman H, Geggel A, Rouhani S, Oehrig J, Baker M (2017). Consequences of large-scale salinity alteration during the *Deepwater Horizon* oil spill on subtidal oyster populations. Marine Ecology Progress Series.

[ref-59] Reddy CM, Arey JS, Seewald JS, Sylva SP, Lemkau KL, Nelson RK, Carmichael CA, McIntyre CP, Fenwick J, Ventura GT, Van Mooy BA (2012). Composition and fate of gas and oil released to the water column during the Deepwater Horizon oil spill. Proceedings of the National Academy of Sciences.

[ref-60] Renner R, Thacker PD, Lubick N, Patel-Predd P, Christen K (2006). Exxon Valdez oil no longer a threat?. Environmental Science & Technology.

[ref-61] Rice SD (1973). Toxicity and avoidance tests with Prudhoe Bay oil and pink salmon fry. International oil spill conference, vol. 1973, no. 1.

[ref-62] Rozas LP, LaSalle MW (1990). A comparison of the diets of Gulf killifish, *Fundulus grandis* Baird and Girard, entering and leaving a Mississippi brackish marsh. Estuaries.

[ref-63] Rozas LP, Martin CW, Valentine JF (2013). Effects of reduced hydrological connectivity on the nursery use of shallow estuarine habitats within a river delta. Marine Ecology Progress Series.

[ref-64] Rozas LP, Minello TJ, Miles MS (2014). Effect of Deepwater Horizon oil on growth rates of juvenile penaeid shrimps. Estuaries and Coasts.

[ref-65] Saaristo M, Brodin T, Balshine S, Bertram MG, Brooks BW, Ehlman SM, McCallum ES, Sih A, Sundin J, Wong BB, Arnold KE (2018). Direct and indirect effects of chemical contaminants on the behaviour, ecology and evolution of wildlife. Proceedings of the Royal Society B: Biological Sciences.

[ref-66] Schaefer J, Frazier N, Barr J (2016). Dynamics of near-coastal fish assemblages following the deepwater horizon oil spill in the Northern Gulf of Mexico. Transactions of the American Fisheries Society.

[ref-67] Schlenker LS, Welch MJ, Mager EM, Stieglitz JD, Benetti DD, Munday PL, Grosell M (2019a). Exposure to crude oil from the deepwater horizon oil spill impairs oil avoidance behavior without affecting olfactory physiology in Juvenile Mahi-Mahi (*Coryphaena hippurus*). Environmental Science & Technology.

[ref-68] Schlenker LS, Welch MJ, Meredith TL, Mager EM, Lari E, Babcock EA, Pyle GG, Munday PL, Grosell M (2019b). Damsels in distress: oil exposure modifies behavior and olfaction in bicolor damselfish (*Stegastes partitus*). Environmental Science & Technology.

[ref-69] Schrope M (2010). The lost legacy of the last great oil spill. Nature News.

[ref-70] Seuront L (2010). Zooplankton avoidance behaviour as a response to point sources of hydrocarbon-contaminated water. Marine and Freshwater Research.

[ref-71] Silliman BR, Van de Koppel J, McCoy MW, Diller J, Kasozi GN, Earl K, Adams PN, Zimmerman AR (2012). Degradation and resilience in Louisiana salt marshes after the BP–Deepwater Horizon oil spill. Proceedings of the National Academy of Sciences of the United States of America.

[ref-72] Smultea MA, Würsig B (1995). Behavioral reactions of bottlenose dolphins to the Mega Borg oil spill, Gulf of Mexico 1990. Aquatic Mammals.

[ref-73] Stieglitz JD, Mager EM, Hoenig RH, Benetti DD, Grosell M (2016). Impacts of Deepwater Horizon crude oil exposure on adult mahi-mahi (*Coryphaena hippurus*) swim performance. Environmental Toxicology and Chemistry.

[ref-74] Turner RE, McClenachan G, Tweel AW (2016). Islands in the oil: quantifying salt marsh shoreline erosion after the Deepwater Horizon oiling. Marine Pollution Bulletin.

[ref-75] Vastano AR, Able KW, Jensen OP, López-Duarte PC, Martin CW, Roberts BJ (2017). Age validation and seasonal growth patterns of a subtropical marsh fish: the Gulf Killifish, *Fundulus grandis*. Environmental Biology of Fishes.

[ref-76] Webster MM, Atton N, Ward AJ, Hart PJ (2007). Turbidity and foraging rate in threespine sticklebacks: the importance of visual and chemical prey cues. Behaviour.

[ref-77] Weis JS, Smith G, Zhou T, Santiago-Bass C, Weis . (2001). Effects of Contaminants on Behavior: biochemical Mechanisms and Ecological Consequences: killifish from a contaminated site are slow to capture prey and escape predators; altered neurotransmitters and thyroid may be responsible for this behavior, which may produce population changes in the fish and their major prey, the grass shrimp. Bioscience.

[ref-78] Whitehead A, Dubansky B, Bodinier C, Garcia TI, Miles S, Pilley C, Raghunathan V, Roach JL, Walker N, Walter RB, Rice CD (2012). Genomic and physiological footprint of the Deepwater Horizon oil spill on resident marsh fishes. Proceedings of the National Academy of Sciences of the United States of America.

[ref-79] Zengel S, Montague CL, Pennings SC, Powers SP, Steinhoff M, Fricano G, Schlemme C, Zhang M, Oehrig J, Nixon Z, Rouhani S (2016). Impacts of the *Deepwater Horizon* oil spill on salt marsh periwinkles (*Littoraria irrorata*). Environmental Science & Technology.

